# Novel Nuclear Localization and Potential Function of Insulin-Like Growth Factor-1 Receptor/Insulin Receptor Hybrid in Corneal Epithelial Cells

**DOI:** 10.1371/journal.pone.0042483

**Published:** 2012-08-03

**Authors:** Yu-Chieh Wu, Meifang Zhu, Danielle M. Robertson

**Affiliations:** Department of Ophthalmology, The University of Texas Southwestern Medical Center, Dallas, Texas, United States of America; UC Berkeley, United States of America

## Abstract

**Background:**

Type I insulin-like growth factor receptor (IGF-1R) and insulin receptor (INSR) are highly homologous molecules, which can heterodimerize to form an IGF-1R/INSR hybrid (Hybrid-R). The presence and biological significance of the Hybrid-R in human corneal epithelium has not yet been established. In addition, while nuclear localization of IGF-1R was recently reported in cancer cells and human corneal epithelial cells, the function and profile of nuclear IGF-1R is unknown. In this study, we characterized the nuclear localization and function of the Hybrid-R and the role of IGF-1/IGF-1R and Hybrid-R signaling in the human corneal epithelium.

**Methodology/Principle Findings:**

IGF-1-mediated signaling and cell growth were examined in a human telomerized corneal epithelial (hTCEpi) cell line using co-immunoprecipitation, immunoblotting and cell proliferation assays. The presence of Hybrid-R in hTCEpi and primary cultured human corneal epithelial cells was confirmed by immunofluorescence and reciprocal immunoprecipitation of whole cell lysates. We found that IGF-1 stimulated Akt and promoted cell growth through IGF-1R activation, which was independent of the Hybrid-R. The presence of Hybrid-R, but not IGF-1R/IGF-1R, was detected in nuclear extracts. Knockdown of INSR by small interfering RNA resulted in depletion of the INSR/INSR and preferential formation of Hybrid-R. Chromatin-immunoprecipitation sequencing assay with anti-IGF-1R or anti-INSR was subsequently performed to identify potential genomic targets responsible for critical homeostatic regulatory pathways.

**Conclusion/Significance:**

In contrast to previous reports on nuclear localized IGF-1R, this is the first report identifying the nuclear localization of Hybrid-R in an epithelial cell line. The identification of a nuclear Hybrid-R and novel genomic targets suggests that IGF-1R traffics to the nucleus as an IGF-1R/INSR heterotetrameric complex to regulate corneal epithelial homeostatic pathways. The development of novel therapeutic strategies designed to target the IGF-1/IGF-1R pathway must take into account the modulatory roles IGF-1R/INSR play in the epithelial cell nucleus.

## Introduction

The type 1 insulin-like growth factor receptor (IGF-1R) belongs to the receptor tyrosine kinase (RTK) superfamily and mediates crucial signaling pathways which function to regulate a variety of biological responses, including anchorage-dependent/independent cell growth, proliferation, differentiation, and apoptosis [1]. Stimulated by ligands (insulin like growth factors, IGFs) at the plasma membrane, signaling events mediated by the IGF-1R are primarily through activation of phosphatidylinositol 3-kinase (PI3K)-Akt and mitogen-activated protein kinase (MAPK) pathways.

Insulin and IGF-1 receptors share 60% overall amino acid sequence homology and 84% homology in their tyrosine kinase (TK) domains. Insulin receptor (INSR) and IGF-1R exist as heterotetramers linked by disulfide bonds, consisting of two ligand-binding, extracellular α subunits and two β subunits that span the plasma membrane via a transmembrane domain. The intracellular TK domain of the β subunit becomes transphosphorylated by the dimeric subunit partner after ligand binding [2]. IGF-1R and INSR can heterodimerize to form IGF-1/insulin hybrid receptor (Hybrid-R), which is composed of one α- and one β-subunit of each receptor. The ligands of these receptors, IGFs (IGF-1 and IGF-2) and insulin, also show high sequence similarity. Collectively, the presence of Hybrid-R and high homology between the receptors and between their ligands results in cross-talk between IGF-1 and insulin signaling [3]. The ligands for the Hybrid-R however, have been controversially discussed, and the binding affinity of IGFs and insulin to the Hybrid-R appears to be cell-type specific [3], [4]. Although IGF-1R and INSR share strong homologies, the homodimeric IGF-1R/IGF-1R and INSR/INSR have different cellular functions: IGF-1R signaling is principally involved in regulating cell growth, whereas INSR signaling regulates carbohydrate metabolism [5].

In the human corneal epithelium *in situ*, the IGF-1R has been previously shown to localize to the plasma membrane and cytoplasm of human corneal and conjunctival epithelium [6]. IGF-1R has also been recently shown to translocate to the nucleus of human tumor cells in a ligand-dependent manner [7]. Importantly, once inside the nucleus, IGF-1R has been shown to function as a transcriptional co-factor; however, to date, no specific targets have been identified [8]. The nuclear localization of other RTK family members has been reported for the epidermal growth factor receptor (EGFR) and fibroblast growth factor receptor (FGFR), where these receptors have been shown to function as transcription factors that regulate genes involved in control of the cell cycle [9], [10], [11], [12], [13]. Similar to previous studies, our recent data demonstrate the nuclear localization of IGF-1R [14] in a corneal epithelial cell line and in primary cultured human corneal epithelium. Due to the presence of the IGF-1R detected in the chromatin bound fraction of corneal epithelial cells, we speculate that the IGF-1R plays a direct role in gene regulation [14], which is of great significance for further investigation.

In this study, we characterized the presence of IGF-1R/INSR heterodimers in a human corneal epithelial cell line (hTCEpi) and have shown that IGF-1, but not insulin, can activate IGF-1R/INSR hybrid phosphorylation. Moreover, IGF-1 induces proliferation through the IGF-1R/PI3K/Akt signaling pathway in corneal epithelial cells, and not through Hybrid-R signaling. More importantly, we provide novel evidence that Hybrid-R instead of homodimeric IGF-1R/IGF-1R exists in the corneal epithelial nucleus, where preferential formation of Hybrid-R was found. We further provide data supporting that nuclear IGF-1R targets potential genes associated with cell adhesion, proliferation and survival pathways, suggesting a novel role for the nuclear Hybrid-R in mediating homeostatic events in the corneal epithelium.

## Materials and Methods

### Cell Isolation and Culture

Human telomerized corneal epithelial cells (hTCEpi), previously published and characterized in detail [15], were routinely maintained in serum-free keratinocyte growth media (KGM-2, Lonza, Walkersville, MD) containing 0.15 mM calcium and supplemented with 0.4% bovine pituitary extract, 0.1% human epidermal growth factor, 0.1% insulin, 0.1% hydrocortisone, 0.1% transferrin, 0.1% epinephrine and 0.1% gentamicin sulfate amphotericin B. For primary corneal epithelial cultures, human donor eye bank corneas (Tissue Transplant Services, UT Southwestern Medical Center, Dallas, TX) were first digested in 5U/ml dispase (Invitrogen, Carlsbad, CA) overnight at 4°C. Intact epithelial cell layers were then carefully peeled off and underwent a second digestion in dispase for 2 h at 37°C. After washing with CnT-20 medium (Zen Bio, Research Triangle Park, NC), individual cells were separated by gentle pipetting and seeded onto collagen IV coated dishes (Biocoat, BD Biosciences, San Jose, CA). Cells were grown in CnT-20 cell culture media enriched for progenitor cell culture and used for experiments between passages 2 to 4. All cells were incubated at 37°C in 5% CO_2_.

### Antibodies

Antibodies used for neutralization, immunoprecipitation, immunofluorescence, and immunoblotting included mouse monoclonal anti-Akt1, rabbit polyclonal anti-phospho-serine 473 Akt1, rabbit polyclonal anti-GAPDH, rabbit polyclonal anti-insulin receptor β (C-19), mouse monoclonal anti-insulin receptor α (MA-20), mouse monoclonal anti-insulin receptor β (CT-3), and mouse monoclonal anti-phospho-tyrosine (PY20) from Santa Cruz Biotechnology (Santa Cruz, CA); rabbit polyclonal anti-IGF-1Rβ (CST#3027), rabbit monoclonal anti-phospho-IGF-1Rβ (Tyr1135), and rabbit anti-phospho-IGF-1Rβ (Tyr1131)/INSRβ (Tyr1146) (Cell Signaling, Danvers, MA); mouse monoclonal anti-IGF-1Rα (24–31) (Abcam, Cambridge, MA); mouse monoclonal anti-IGF-1Rα (αIR3) from EMD Chemicals (Gibbstown, NJ); rabbit polyclonal anti-SP1 (Millipore, Temecula, CA); and mouse monoclonal anti-β-actin (Sigma, St. Louis, MO).

### Ligand Stimulation, IGF-1R Neutralization, and IGF-1R/PI3K/Akt Inhibition

Cells (3×10^5^) were seeded in 6-well plates and grown overnight. The second day, cells were starved in keratinocyte basal media (KBM-2, Lonza, Walkersville, MD) for 24 h and then stimulated with 100 ng/ml of recombinant human IGF-1 (BioVision Inc, Mountain View, California) or insulin (Sigma, St. Louis, MO) for 15 min. For IGF-1R neutralization, after starvation cells were first treated with 5 µg/ml of αIR3 or control mouse IgG for 4 h and then stimulated with 100 ng/ml of IGF-1 for 15 min. For PI3K inhibition, starved cells were treated with 50 µM of LY294002 (EMD Chemicals, Gibbstown, NJ) for 1 h and then stimulated with 100 ng/ml of IGF-1 for 15 min. Immediately after ligand stimulation, cell lysates were harvested and stored at −20°C for further analysis.

### Cell Lysis and Immunoblotting

For collection of whole cell lysates, cells were washed with cold phosphate buffered saline (PBS) and then lysed directly in the culture dish using radioimmunoprecipitation assay (RIPA) buffer (25 mM Tris·HCl pH 7.6, 150 mM NaCl, 1% NP-40, 1% sodium deoxycholate, 0.1% SDS) containing Halt™ protease and phophatase inhibitor single-use cocktail (Thermo Fisher, Rockford, IL) on ice for 5 min. Lysates were gathered using a cell scraper and then collected into the microcentrifuge tubes. Cell debris was removed by centrifugation of cell lysates at 14,000×g for 15 min. Supernatant of the lysates were boiled for 5 min in 2x SDS-sample buffer (50 mM Tris·HCl, pH 6.8, 10% glycerol, 4% SDS, 0.01% bromophenol blue, 2% β-mercaptoethanol), resolved on a TGX™ precast polyacrylamide gel (Bio-rad, Hercules, CA) and subsequently transferred to an immobilon-P PVDF membrane (Millipore, Temecula, CA). Membranes were blocked in 5% non-fat milk for 30 min at room temperature (RT) and blotted using the indicated primary antibodies overnight at 4°C. Following a 1 h incubation with a peroxidase-conjugated anti-mouse or anti-rabbit secondary antibody (Santa Cruz Biotechnology, Santa Cruz, CA), protein was visualized using ECL Plus Detection Reagents (Amersham Biosciences, Piscataway, NJ) and imaged on a Typhoon Variable Mode Imager.

### Subcellular Fractionation

Cytoplasmic and nuclear extracts were separated using NE-PER nuclear and cytoplasmic extraction reagents (Thermo Fisher, Rockford, IL) according to manufacturer instructions. Briefly, cells were harvested using trypsin-EDTA and subsequently washed with chilled PBS. Cytosolic proteins were first extracted by disrupting cell membranes, followed by centrifugation. Intact nuclei were washed by cold PBS and then lysed with high salt NE-PER buffer. Both cytosolic and nuclear fractions were stored at −80°C for future use. Further cellular fractions were separated using a protein subcellular fractionation kit (Thermo Fisher, Rockford, IL) according to instructions provided by the manufacturer. Controls for cross-contamination of individual fractions were confirmed by immunoblotting with antibodies against GAPDH (cytosolic extract) and SP1 (nuclear extract).

### Immunoprecipitation

For immunoprecipitation assays, whole cell lysates of 10^6^ cells (∼1 mg) or cytosolic fractions (∼900 µg) were incubated with 1 µg of rabbit polyclonal anti-IGF-1Rβ (CST#3027), rabbit polyclonal anti-INSRβ (C-19), mouse monoclonal anti-INSRα (MA-20), or mouse monoclonal anti-IGF-1Rα (αIR3) with continuous rocking overnight at 4°C. Nuclear fractions prepared with NE-PER extraction reagents were diluted 1∶2 with RIPA buffer containing protease and phophatase inhibitor cocktail and then split into half. Equal amounts of the nuclear fractions (∼400 µg) were incubated with 1 µg of anti-IGF-1Rα (αIR3) or anti-INSRβ (C-19) as explained above. 30 µl of immobilized protein A plus (Thermo Fisher, Rockford, IL) was then added to the antigen-antibody complexes with continuous rocking for 2 h at RT. Following centrifugation, the pellet was washed 5 times with cold PBS. 25 µl of 4x sample buffer was added to resuspend the resulting pellet and boiled for 5 minutes. Immunoprecipitated lysates were then immunoblotted as described above. For reducing immunoprecipitation, whole cell lysates were treated with 1 mM of dithiothreitol (DTT) at pH 8.5 for 30 min at RT to break up the class 1 disulfide bond holding the two receptor αβ-dimers together. The reduction was then terminated by adding 3 mM of *N*-ethylmaleimide (NEM) at pH 7.5 [16]. pH was adjusted with 1M of Tris·Base (pH 10) or Tris·HCl (pH 4) and confirmed by pH test strips (Sigma, St. Louis, MO). After reduction, cell lysates were split into half and then immunoprecipitated with either anti-IGF-1Rβ (CST#3027) or anti-IRβ (C-19) as described above.

### Immunofluorescence

For immunofluorescent studies, cells were seeded onto 12 mm glass coverslips and allowed to adhere overnight. Cells were first rinsed with PBS and then fixed in 3% paraformaldehyde (Electron Microscopy Sciences, Fort Washington, PA) in PBS for 10 min. After 3 washes with PBS, cells were permeabilized in 0.2% Triton X-100 for 10 min and then blocked using 0.5% bovine serum albumin (Sigma, St. Louis, MO) for 30 min. Samples were then incubated in primary antibody at 4°C overnight and subsequently washed in PBS and stained with an Alexa Fluor 488 (Cell Signaling, Danvers, MA), Dylight 549 or Dylight 649 (GeneTex Inc., Irving, CA) secondary antibody for 1 h at RT. Nuclei were counterstained with Propidium Iodide (PI). All samples were mounted on slides using Prolong gold antifade reagent (Invitrogen, Carlsbad, CA) and imaged on a Leica SP2 laser scanning confocal microscope (Leica Microsystems, Heidelberg, Germany) using a 63x water objective. All images were sequentially scanned to avoid spectral crosstalk between channels. After deconvolution by AutoQuant X (Media Cybernetics, Inc., Bethesda, MD), surface rendering and co-localization analysis were performed utilizing Imaris 7.3.2 (Bitplane Inc., Saint Paul, MN) via three-dimensional volume rendering of z-stacks after background correction. The level of co-localization in the three-dimensional volume was measured as percent of volume of the channel above threshold co-localized. A second measure of the intensity of co-localization between two signals was obtained by calculating the correlation between the intensities of the co-localized three-dimensional pixels (Pearson’s correlation coefficient). The slope in the two-dimensional histogram derived from the least-square fit is directly proportional to the Pearson correlation coefficient. The two-dimensional histogram was obtained after the software automatically selected the co-localized areas.

### Cell Proliferation Assay

To evaluate cell proliferation, 5×10^3^ cells were seeded in 96-well plates and starved in KBM-2 media for 24 h. Cells were cultured for 4 days in KBM-2 media without cytokine (control) or with IGF-1 or insulin, without or with the anti-IGF-1Rα (αIR3) or recombinant human IGFBP-3 (Sino Biological, Beijing, China). At the end of culture, cellular DNA content was measured via fluorescent dye binding with a CyQUANT NF Cell Proliferation Assay Kit (Invitrogen, Carlsbad, CA). Fluorescent signals were detected using a Synergy 2 Multi-Mode Microplate Reader (BioTek, Winooski, VT) with excitation at 485 nm and emission at 530 nm. Values are given as mean and standard deviation with potential differences analyzed with Student’s *t*-test. *P*<0.05 was considered significant.

### SiRNA Knockdown

Gene-specific knockdown in hTCEpi cells was performed by transfecting the cells with annealed, double-stranded small inhibitory RNA. hTCEpi cells were seeded at 50% confluence into 100-mm dishes before transfection. The second day, 72 pmol of negative control siRNA, INSR siRNA (GeneSolution siRNA, Qiagen, Valencia, CA), or 12 µl of RNAiMAX reagents (Invitrogen, Carlsbad, CA) were mixed with 600 µl of antibiotic-free KBM-2 and incubated at RT for 5 min. siRNA and transfection reagents were combined and incubated at RT for 20 min. Transfection mixture was added into the hTCEpi cell culture containing 6 ml of antibiotic-free KBM-2 and incubated for a total of 48 to 72 h. The target sequences of siRNA duplexes used in this study were listed in Table 1.

**Table 1:** GeneSolution^a^ siRNA target sequences.

	Catalog number	Target sequences
IGF-1R	SI02624552	TCGAAGAATCGCATCATCATA
	SI03096926	CTGGACTCAGTACGCCGTTTA
	SI00017521	ATGGAGAATAATCCAGTCCTA
	SI02624545	AGGATTGAGTTTCTCAACGAA
INSR	SI00004522	ACCGCTTTACGCTTCTTCAAA
	SI03115434	TCCGGGTACCGCGAAGGGCAA
	SI00004508	TCGAACGATGTTGGACTCATA
	SI00004515	CAACGGGAGTCTGATCATCAA
Negative control	1022076	AATTCTCCGAACGTGTCACGT

a GeneSolution contains equal mixture of four siRNA duplexes.

### ChIP-seq Assay

Chromatin-immunoprecipitation was performed using Pierce Agarose ChIP Kit (Thermo Fisher, Rockford, IL) according to manufacturer’s instruction. Briefly, confluent hTCEpi cells cultured in KGM-2 media in 100-mm dishes were crosslinked by 1% formaldehyde at RT for 10 min. The reaction was stopped by incubation with 250 mM of glycine at RT for 5 min. After rinsing twice with ice-cold PBS, cells were scraped and collected in ice-cold PBS containing Halt™ protease and phophatase inhibitor single-use cocktail. Cells were pelleted and lysed by dissolving the cell membrane to obtain intact nuclei. Nuclei were treated with 25 U/ml of micrococcal nuclease to digest genomic DNA and then the nuclear fractions containing sheared DNA fragments were collected and subject to immunoprecipitation. 10% of total input DNA fragments were saved for negative background control. DNA fragments were further immunoprecipitated with antibodies against IGF-1R (CST#3027) or INSR (C-19). Enriched IGF-1R or INSR-bound DNA complexes were treated with proteinase K to remove proteins from DNA preparations. DNA fragments were further purified through DNA clean-up columns. Purified DNA samples were subjected to massively parallel DNA sequencing on ABI 5500 XL SOLiD sequencers.

### Bioinformatics Analysis and Databases

Mapping of ChIP-Seq reads was done with the LifeScope package. For general mapped file manipulation, Picard and SAMtools were used to sort, remove, and duplicate the sequence reads [17]. Filtered reads were aligned to the human reference genome (hg19) form UCSC. The aligned reads were passed to MACS [18] and PeakRanger [19] for peak calling, with the total input DNA background used in each comparison. MACS and PeakRanger software were used to find regions (peaks) with statistically significant enrichment with the default P value cutoff of 10^−5^. The final peaks were assigned to BEDTools package to identify nearest regulated genes information [20]. Functional annotation of the nearest genes was retrieved from DAVID bioinformatics database [21], [22] (http://david.abcc.ncifcrf.gov; release 6.7), using GENBANK_ACCESSION as the main identifier. P-value was assessed by a corrected hyper geometric test (DAVID’s EASE score; modified Fisher Exact p-value); the more enriched terms were associated with the smaller p-value.

## Results

### IGF-1 Activates IGF-1R and INSR Phosphorylation and Akt Signaling

**Figure 1:**
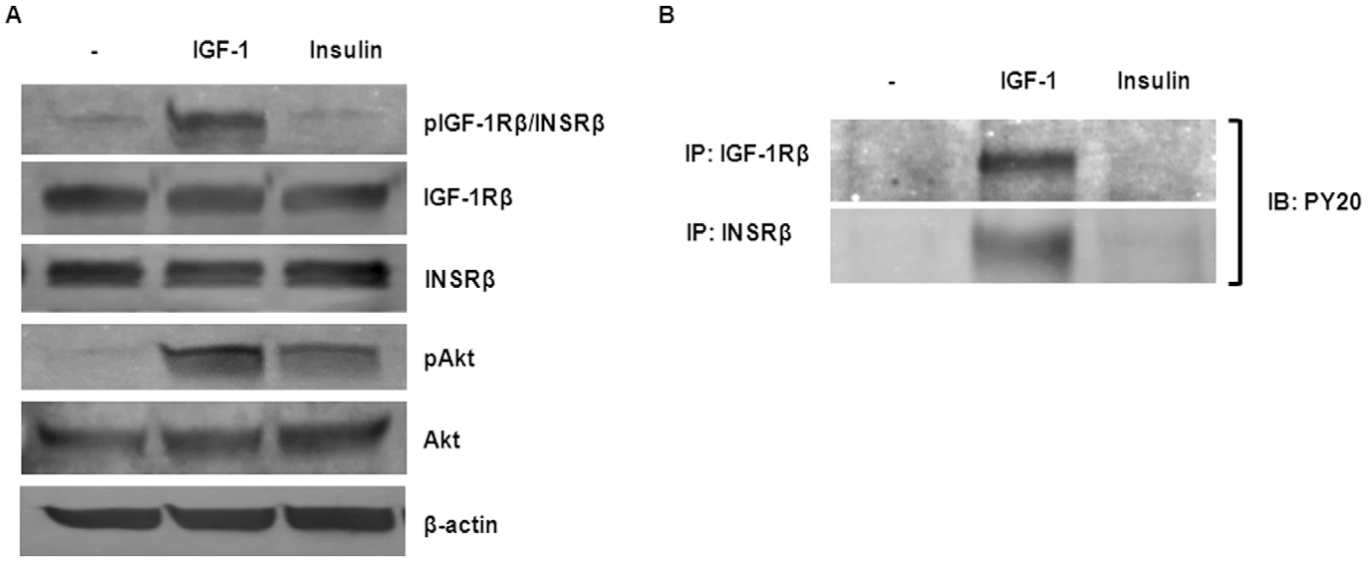
IGF-1 induces the canonical IGF-1R/Akt signaling pathway. (A) Akt phosphorylation induced by IGF-1 and insulin. hTCEpi cells were starved for 24 h in KBM-2 culture medium and then stimulated with IGF-1 (100 ng/ml) or insulin (100 ng/ml) for 15 min. Whole cell lysates were immunoblotted (IB) with anti-phospho-IGF-1Rβ, anti-IGF-1Rβ, anti-INSRβ, anti-phospho-Akt, anti-Akt, or anti-β-actin (loading control) as indicated. (B) IGF-1-induced IGF-1R phosphorylation. Protein lysates obtained as above were immunoprecipitated (IP) with anti-IGF-1Rβ (CST#3027) or anti-INSRβ (C-19) and phosphorylation of both receptors was detected on immunoblots with anti-PY20. Blots are representative of at least two repeated experiments.

To examine the response of IGF-1R and INSR to IGF-1 and insulin, hTCEpi cells were starved for 24 h and incubated with 100 ng/ml of IGF-1 or insulin for 15 min. Whole cells lysates were then analyzed by immunoblotting with antibodies against phospho-IGF-1Rβ and phospho-Akt (Fig. 1A). IGF-1 stimulated IGF-1R and Akt phosphorylation while insulin at the same concentration only simulated a modest level of Akt activation. We were unable to detect IGF-1R or INSR phosphorylation induced by insulin. It is likely that insulin has very low effect on either IGF-1R or INSR activation in our cell line at this time point. To further characterize the ligand effect on receptor phosphorylation, whole cell lysates described above were first immunoprecipitated with anti-IGF-1R or anti-INSR, followed by immunoblot analysis with anti-phospho-tyrosine (Fig. 1B). Only IGF-1 stimulation appeared to activate IGF-1R and INSR phosphorylation; however, IGF-1 was unable to stimulate INSR:INSR phosphorylation (Fig. S1). Collectively, these findings suggest that IGF-1 may activate INSR phosphorylation through the IGF-1R/INSR hybrid.

### Human Corneal Epithelium Express IGF-1R/INSR Hybrid (Hybrid-R)

**Figure 2:**
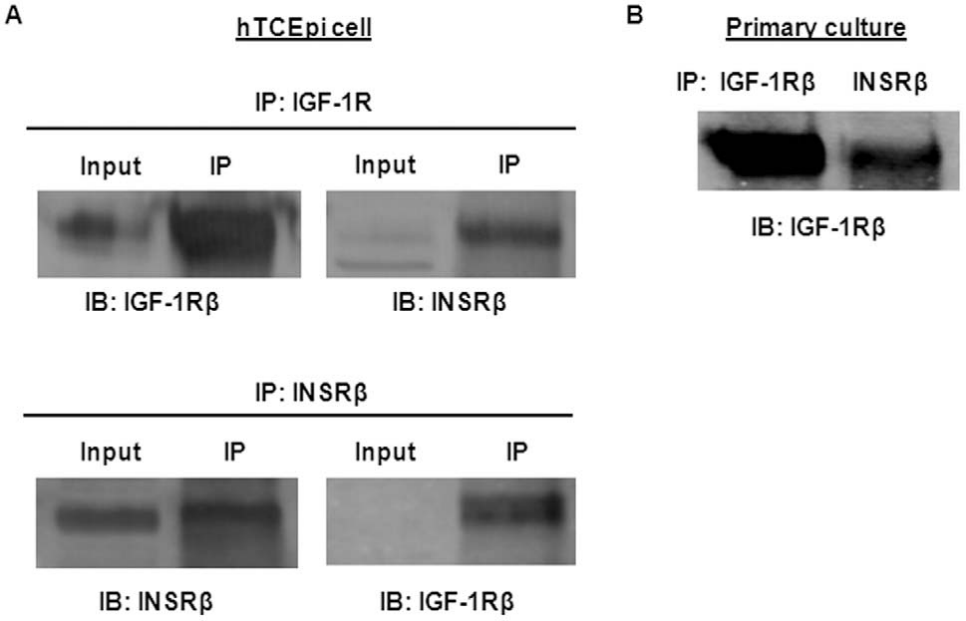
Presence of IGF-1 receptor and insulin receptor (IGF-1R/INSR) hybrid in corneal epithelium. (A) Reciprocal immunoprecipitation (IP) of IGF-1 receptor and insulin receptor in the hTCEpi cell line. Whole cell lysates were subjected to IP with antibodies against either the IGF-1 receptor (IGF-1R) β-subunit (CST#3027) or the insulin receptor (INSR) β-subunit (C-19). Immunoprecipitated products were then immunoblotted with the same antibodies. Input represents the whole cell lysates before IP. (B) Co-precipitation of IGF-1R/INSR hybrid in primary corneal epithelial cell cultures. Protein lysates from passage 3 of human corneal epithelial cells were equally immunoprecipitated with anti-IGF-1Rβ (CST#3027) or anti-INSRβ (C-19), and detection of IGF-1R/IGF-1R or IGF-1R/INSR was performed with immunoblots with the same antibody against IGF-1Rβ.

To ascertain whether hTCEpi cells express Hybrid-R, we performed a reciprocal immunoprecipitation of whole cell lysates using either anti-IGF-1R or anti-INSR and then probed with antibodies against either anti-INSR or anti-IGF-1R. As shown in Fig. 2A, IGF-1R co-precipitated with INSR, and a significant amount of INSR co-precipitated with IGF-1R. Because the IGF-1R and INSR antibodies used here do not cross-react with INSR or IGF-1R, respectively, our data suggest the presence of Hybrid-R in cultured corneal epithelium. To further establish the existence of Hybrid-R in the corneal epithelium, protein lysates from primary cultured human donor corneal epithelial cells were immunoprecipitated with anti-IGF-1R or anti-INSR. The immunoprecipitation products were immunoblotted with anti-IGF-1R and the blot showed the co-precipitation of IGF-1R with INSR in primary cells (Fig. 2B). Our data support the physiological presence of Hybrid-R in human corneal epithelium.

### IGF-1 Activates Hybrid-R Phosphorylation

**Figure 3:**
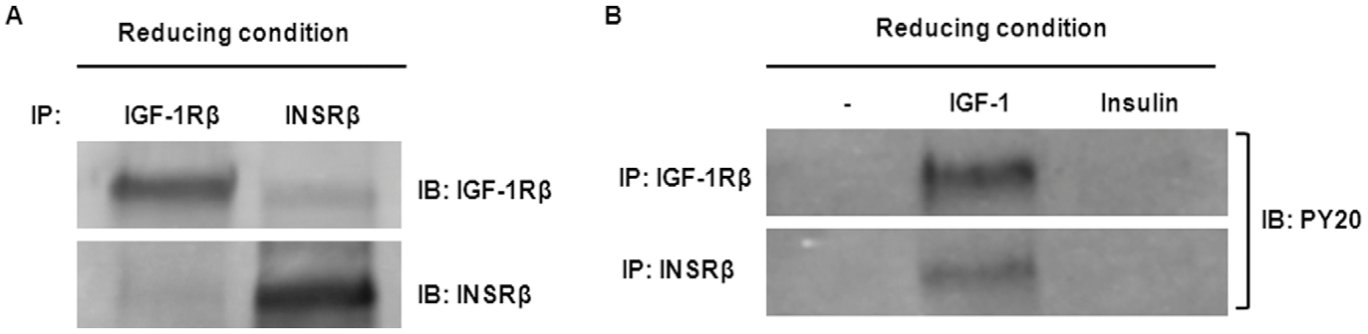
IGF-1 activates the IGF-1R/INSR hybrid. (A) Existence of IGF-1R and INSR αβ-dimers after reducing immunoprecipitation. hTCEpi lysates were reduced with DTT to break the receptors into αβ-dimers. Reduced lysates were then immunoprecipitated with rabbit polyclonal anti-IGF-1Rβ (CST#3027) or anti-INSRβ (C-19). *Top panel*: Immunoblot of INSRβ immunoprecipitates from reduced lysates with anti-IGF-1Rβ showed no or faint bands corresponding to the IGF-1Rβ. *Bottom panel*: Immunoblot of IGF-1Rβ immunoprecipitates from reduced lysates with anti-INSRβ showed no or faint bands corresponding to the INSRβ. (B) Activation of IGF-1R and INSR in hTCEpi cells by IGF-1 stimulation. Protein lysates of hTCEpi cells after stimulation by IGF-1 (100 ng/ml) or insulin (100 ng/ml) for 15 min were collected and reduced by DTT and then subjected to reducing IP. IGF-1, but not insulin, induced phosphorylation of IGF-1 receptor (*Top panel*) and insulin receptor (*Bottom panel*) detected on immunoblots with anti-PY20.

To clarify the stimulation effect of IGF-1 on INSR phosphorylation, a reducing immunoprecipitation was performed using DTT to reduce the disulfide bonds linking the two αβ-heterodimers of the receptors (Fig. 3). When immunoprecipitating and immunoblotting the reduced lysates with antibodies against IGF-1R, a band corresponding to the IGF-1R was found (Fig. 3A, *top left*), while immunoblotting with anti-INSR showed no or faint INSR band (Fig. 3A, *bottom left)*. Reversely, reduced samples immunoprecipitated with antibodies against INSR, followed by immunoblotting with either anti-INSR or anti-IGF-1R, demonstrated a similar finding with a band corresponding to the INSR and no or faint IGF-1R band (Fig. 3A, *top and bottom, right*). This indicates that the co-precipitation shown in Fig. 2 was not due to cross-reactivity of the antibodies used for IP or that the receptors were kept together by membrane fragments. To specifically detect phosphorylated receptor αβ-dimers, whole cell lysates obtained as described in Fig. 1A were reduced in DTT and then immunoprecipitated with either anti-IGF-1R or anti-INSR. Immunoblots using anti-PY20 showed that IGF-1 induced phosphorylation of both IGF-1Rβ and INSRβ (Fig. 3B). To further examine the effect of IGF-1 on homodimeric INSR phosphorylation, hTCEpi cells were starved for 24 h and incubated with 100 ng/ml of IGF-1 or insulin for 15 min. Whole cells lysates were first immunoprecipitated with anti-INSR (MA-20, which only reacts with INSR/INSR homodimer [23]), followed by immunoblot analysis with anti-phospho-tyrosine (Fig. S1B). Using this antibody (MA-20), the homodimeric INSR was not phosphorylated in response to IGF-1. Taken together, these results suggest that Hybrid-R responds to IGF-1 stimulation in corneal epithelial cells.

### IGF-1 Promotes Corneal Epithelial Cell Growth Through the IGF-1/IGF-1R/PI3K/Akt Signaling Pathway

**Figure 4:**
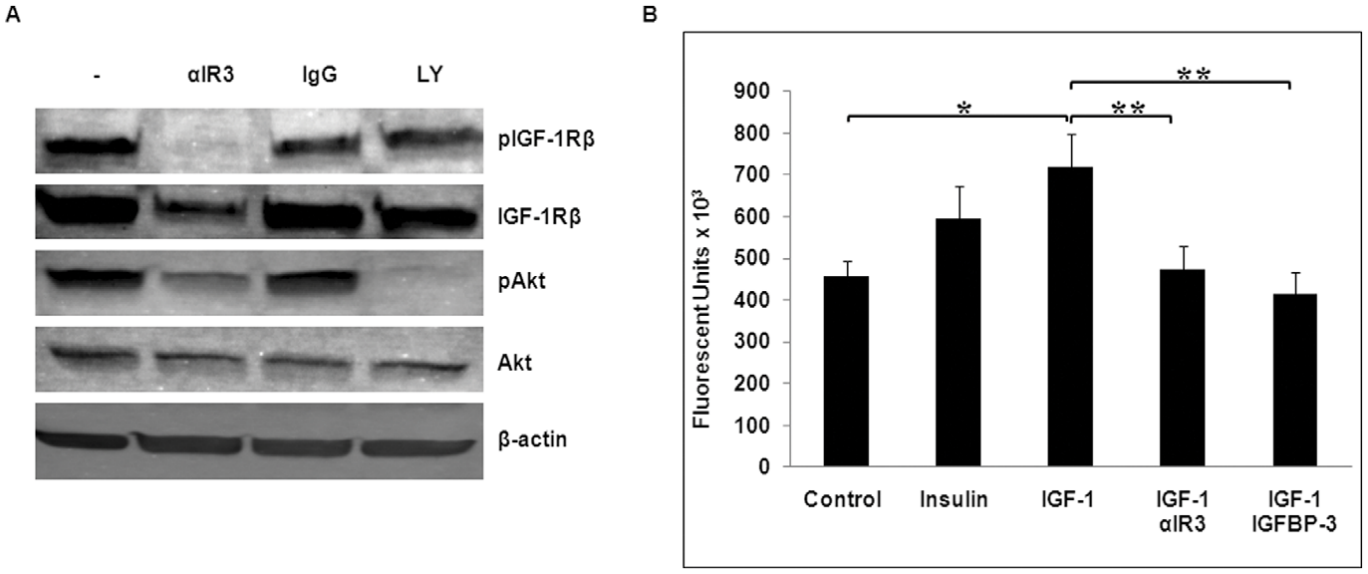
IGF-1 promotes hTCEpi cell proliferation. (A) Neutralization of IGF-1 receptor and inhibition of IGF-1R/PI3K/Akt pathway. hTCEpi cells were starved for 24 h and then cultured with 5 µg/ml of IGF-1R neutralizing antibody (αIR3) or mouse IgG (negative control) for 4 h or with 50 µM of PI3K inhibitor (LY294002, denoted by LY) for 1 h, followed by stimulation with IGF-1 (100 ng/ml) for 15 min. Whole cell lysates were immunoblotted with antibodies as indicated. Antibody against β-actin was used as a loading control. (B) IGF-1 promoted the growth of hTCEpi cells. hTCEpi cells were starved for 24 h and then cultured with 10 ng/ml of IGF-1 or insulin in the absence or presence of either αIR3 (1 µg/ml) or IGFBP-3 (2 µg/ml) for 4 days. Total DNA content was then assayed. Results are the mean fluorescent signal ± s.e. of three independent experiments. *The mean value was significantly different from that obtained in the control group (P<0.05); **the mean fluorescent signal with αIR3 or IGFBP-3 was significantly lower than that with IGF-1 (P<0.05); the mean value with insulin was not significantly higher than that obtained in the control group.

Since we observed Akt activation by IGF-1 in a corneal epithelial cell line, we questioned whether this activation was through direct ligand binding to the receptor. To address this, hTCEpi cells were first incubated with the IGF-1R neutralizing antibody, αIR3, for 4 h, followed by simulation with IGF-1. Immunoblots with anti-phospho-IGF-1Rβ and anti-phospho-Akt revealed that blocking the binding of IGF-1 to IGF-1R by αIR3 inhibited IGF-1-induced IGF-1R and Akt phosphorylation. An equal amount of mouse IgG was used as a negative control, which did not repress phosphorylation of IGF-1R and Akt by IGF-1 (Fig. 4A). In addition, αIR3 antibody, an IGF-1 mimetic, also induced IGF-1R down-regulation due to receptor internalization, which has been previously observed in tumor cells [24]. To determine if IGF-1 activates Akt phosphorylation through IGF-1R/PI3K/Akt cascade in corneal epithelial cells, hTCEpi cells were treated with the PI3K inhibitor (LY294002) for 1 h, followed by simultaneous IGF-1 stimulation for the final 15 min. Without affecting IGF-1R phosphorylation, LY294002 treatment completely inhibited Akt phosphorylation (Fig. 4A). Since LY294002 is a reversible inhibitor, removing LY294002 after 1 h of treatment, followed by adding IGF-1 did not show an inhibitory effect by LY294002 on Akt phosphorylation (data not shown). Our data support that IGF-1 induces Akt activation through the canonical IGF-1R/PI3K/Akt pathway. Akt activation is associated with cell survival and proliferation [25], raising the possibility that IGF-1 could promote corneal epithelial cell growth through Akt signaling. To this end, we measured total DNA content of hTCEpi cells stimulated by IGF-1 at a supra-physiological concentration (10 ng/ml) (Fig. 4B). IGF-1 stimulation increased cell proliferation 58 percent compared to non-treated cells, and both IGF-1R neutralizing antibody (competes in IGF-1R binding with IGF-1) and recombinant IGF-1 binding protein-3 (IGFBP-3) (sequesters IGF-1 from binding to IGF-1R) significantly reduced (P<0.05) IGF-1-induced hTCEpi cell growth. Since αIR3 antibody only reacts with IGF-1R/IGF-1R homodimer [26], complete blocking of proliferation by αIR3 indicates that IGF-1-induced corneal epithelial cell proliferation is mediated by the IGF-1R homodimer, not the Hybrid-R.

### IGF-1R Co-localizes with INSR

**Figure 5:**
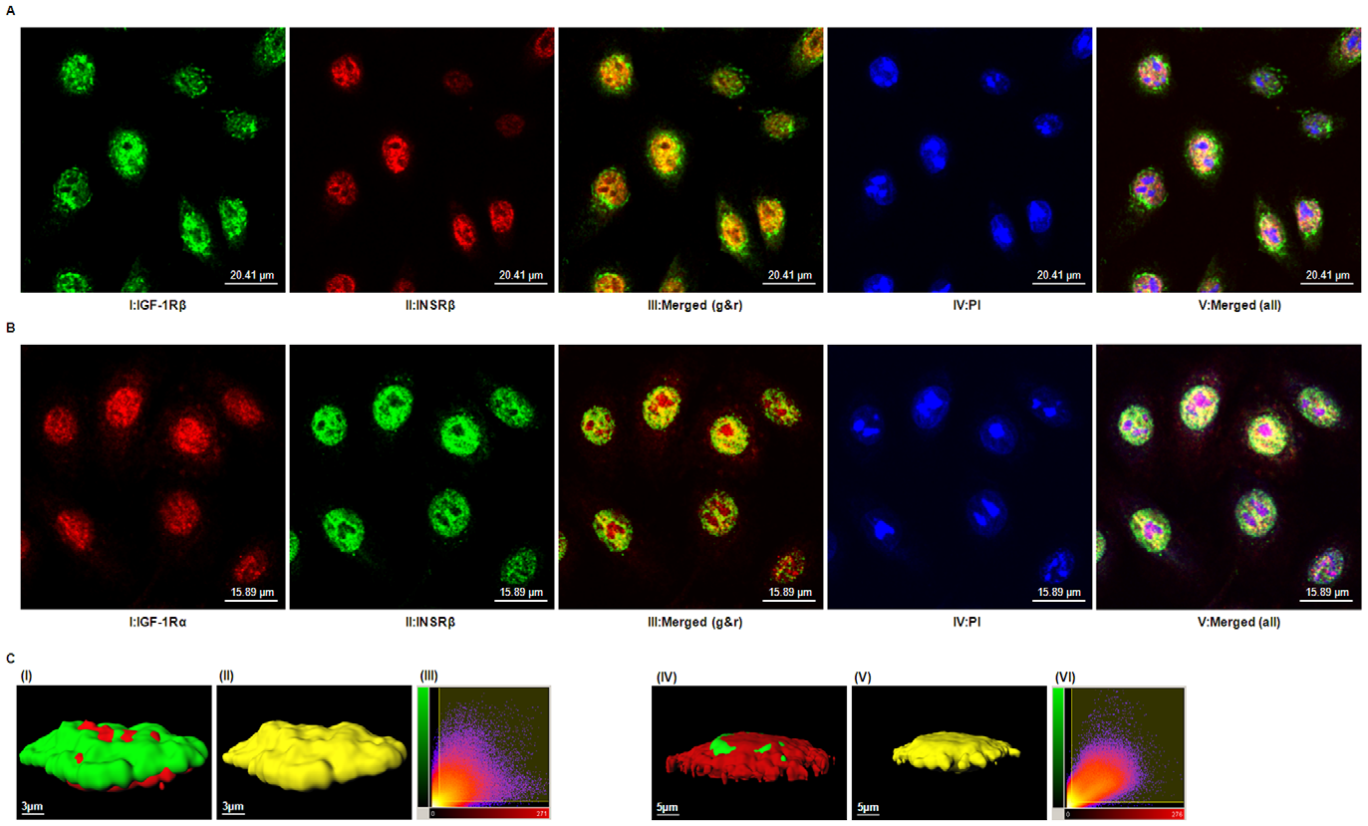
Co-localization of IGF-1R and INSR. (A) Confocal images showing hTCEpi cells labeled with antibodies against IGF-1Rβ (CST#3027) (*panel I*, revealed by *green*), INSRβ (CT-3) (*panel II*, revealed by *red*), or both (*panel III*, merged by panels I and II); nuclei were counterstained with PI (*panel IV*, revealed by *blue*) and overlap of the three channels is shown in *panel V*. Scale: 20.41 µm. (B) Confocal images showing hTCEpi cells labeled with antibodies against IGF-1Rα (24–31) (*panel I*, revealed by *red*), INSRβ (C-19) (*panel II*, revealed by *green*), or both (*panel III*, merged by panels I and II); nuclei were counterstained with PI (*panel IV*, revealed by *blue*) and overlap of the three channels is shown in *panel V*. Scale: 15.89 µm. *Orange or Yellow color* in merged panels III denotes the presence of both IGF-1R and INSR immunoreactivity; *white color* in merged panels V represents the co-localization of both IGF-1R and INSR in the nucleus. (C) Imaris analysis of co-localization. Confocal images of hTCEpi cells labeled with antibodies as described above were further analyzed by Imaris software. The 3D reconstruction of a two-dimensional Z-stack of images illustrates the subcellular distribution of IGF-1Rβ (green) and INSRβ (red) (*Panel I*) or IGF-1Rα (red) and INSRβ (green) (*Panel IV*) in hTCEpi cells. *Panel II and V*, co-localized voxels of panel I and IV, respectively. Scale: 3 or 5 µm. *Panel III or VI* shows the representative two-dimensional histogram of both signals for either panel I or IV. The red signal is on the *x* axis and the green is on the *y* axis. Automatic selected co-localized areas are shown by a rectangular yellow overlay. Data are representative of three independent experiments with 8 to10 cells imaged per experiment.

Triple-labeling experiments with antibodies directed against either the IGF-1R or the INSR and a nuclear counterstain demonstrated the predominant presence of both receptors in the nucleus of hTCEpi cells (Fig. 5). Merged images obtained from confocal microscopy showed significant spatial co-localization of IGF-1Rβ and INSRβ (Fig. 5A) or IGF-1Rα and INSRβ (Fig. 5B), suggesting either distinct IGF-1R and INSR localized to specific cellular domains or the presence of a nuclear Hybrid-R in hTCEpi cells. These co-localization patterns were identical to those seen in primary cultured human corneal epithelium (data not shown). Fig. 5C shows a three-dimensional reconstruction of a Z-stack of two-dimensional images from hTCEpi cells co-stained with IGF-1R and INSR antibodies. Using Imaris analysis software, we calculated the percentage of co-localized voxels (three-dimensional pixels) containing both green and red signals. From this analysis, 37% (IGF-1Rβ and INSRβ) or 38% (IGF-1Rα and INSRβ) of the voxels within the region of interest (ROI) were found to co-localize. To ensure that co-localization results were not affected by the nonspecific binding of fluorescent secondary antibodies, hTCEpi cells were incubated with secondary antibodies followed by confocal imaging. Under these conditions, three-dimensional images from a Z-stack could not be reconstructed due to the absence of co-staining (data not shown). The representative two-dimensional histogram (Fig. 5C, panel III and VI) of each co-staining showed the automatic selected co-localized areas (indicated by a rectangular yellow overlay). Most of the data lying on a diagonal of slope 1 demonstrated a strong co-localization of both signals [27]. Collectively, our data suggest the presence of Hybrid-R in the hTCEpi nucleus.

### Nuclear localization of Hybrid-R

**Figure 6:**
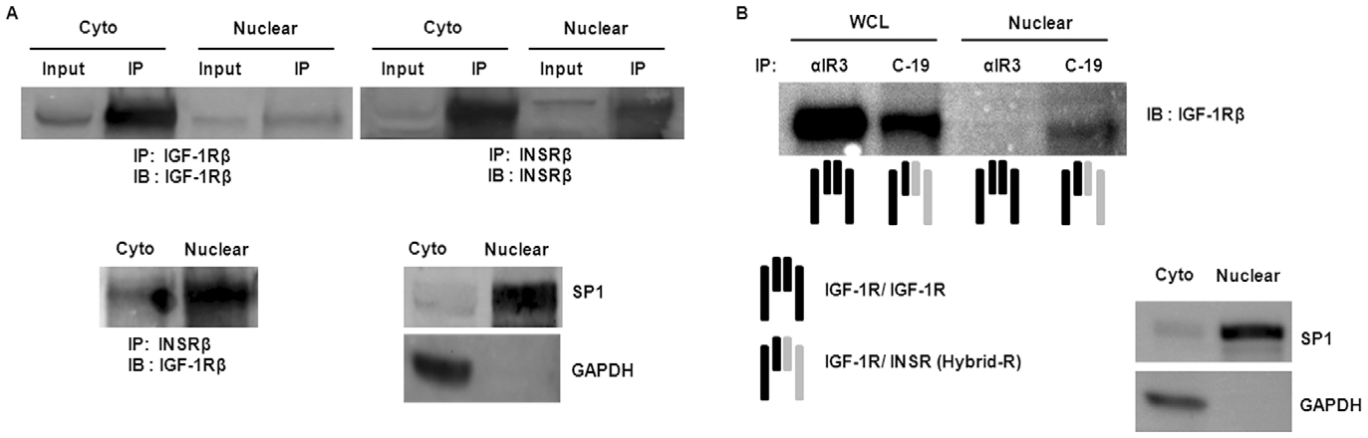
Subcellular localization of IGF-1R/IGF-1R and IGF-1R/INSR hybrid. (A) Presence of IGF-1R/INSR hybrid in the nucleus. Cytosolic (Cyto) and nuclear (Nuclear) fractions of hTCEpi cells were immunoprecipitated with anti-IGF-1Rβ (CST#3027) or anti-INSRβ (C-19) and then immunoblotted with the same antibodies. Bottom left: Immunoblot of IGF-1R immunoprecipitates with anti-INSR indicated the presence of hybrid in the nuclear fraction. (B) Exclusive presence of hybrid in the nucleus. Whole cell lysates (WCL) and nuclear fractions (Nuclear) of hTCEpi cells were subjected to immunoprecipitation with antibodies against IGF-1Rα (αIR3, which reacts with IGF-1R/IGF-1R) or INSRβ (C-19, which reacts with IGF-1R/INSR and INSR/INSR). Immunoprecipitates were then immunoblotted with anti-IGF-1Rβ. Hybrid-R was shown in the WCL and nuclear fractions but IGF-1R/IGF-1R was only detected in the WCL. Controls for cross-contamination between each compartment were confirmed by immunoblotting with antibodies against GAPDH (cytosolic extract) and SP1 (nuclear extract).

To verify the observation of IGF-1R/INSR co-localization, a nuclear immunoprecipitation (IP) was performed (Fig. 6). The purity of both cytosolic and nuclear fractions was confirmed by immunoblotting with marker antibodies (GAPDH for cytosolic fraction and SP1 for nuclear fraction). Cytosolic and nuclear fractions of hTCEpi cell extracts were separated and subjected to reciprocal IP with anti-IGF-1R or anti-INSR, followed by immunoblotting with the same antibodies (Fig. 6A). Hybrid-Rs were detected in both cytosolic and nuclear extracts. We next investigated whether IGF-1R homodimer and/or Hybrid-R was present in the nucleus. To resolve this, nuclear extracts and whole cell lysates were immunoprecipitated with anti-IGF-1Rα (αIR3) or anti-INSR (C-19) to pull-down either IGF-1R/IGF-1R or Hybrid-R, revealed by immunoblotting with anti-IGF-1Rβ (Fig. 6B). Surprisingly, only Hybrid-R was detectable in the nucleus while both IGF-1R/IGF-1R and Hybrid-R were detected in whole cell lysates. The strong co-precipitation of IGF-1Rβ with αIR3 in whole cell lysates suggests a predominantly extranuclear localization of IGF-1R homodimer. Importantly, our findings demonstrate for the first time in any cell system, that nuclear IGF-1R is a Hybrid-R complex.

### Preferential Formation of Hybrid-R in Corneal Epithelial Cells

**Figure 7:**
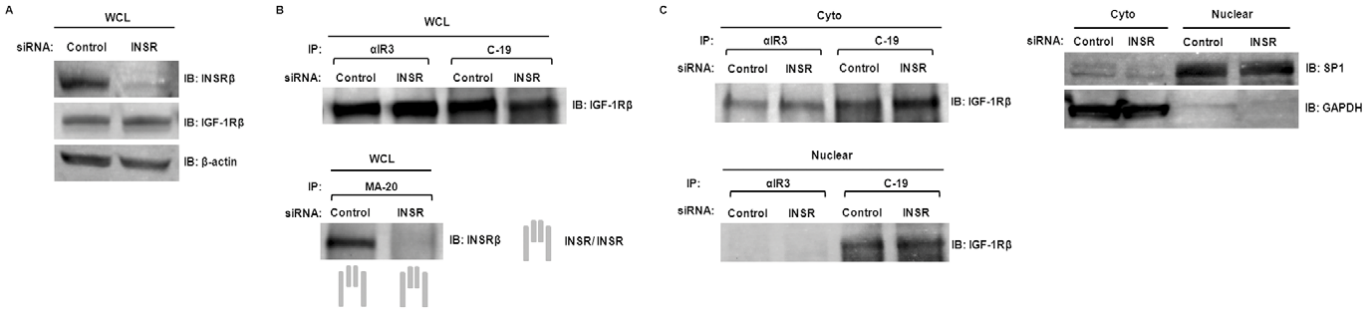
Knockdown of IGF-1R/INSR. Control or INSR siRNA were transfected into hTCEpi cells. Two to three days after transfection, WCL, cytosolic (Cyto) and nuclear fractions (Nuclear) were collected. (A) Immunoblots of WCL with antibodies against INSR (C-19), IGF-1R (CST#3027), or β-actin (loading control). (B) WCL of hTCEpi cells were immunoprecipitated with antibodies αIR3, C-19, or MA-20 (against INSRα and only reacts with INSR/INSR). Immunoprecipitates were then immunoblotted with anti-IGF-1Rβ (CST#3027) or anti-INSRβ (C-19). (C) Cytosolic and nuclear fractions of hTCEpi cells were subjected to immunoprecipitation with antibodies αIR3 or C-19. Immunoprecipitates were then immunoblotted with anti-IGF-1Rβ (CST#3027). GAPDH is the marker for cytosolic extract; and SP1 is the marker for nuclear extract.

We next evaluated the formation of Hybrid-R in response to INSR levels in hTCEpi cells. hTCEpi cells were transfected with INSR-specific siRNA and whole cell lysates were collected and analyzed by immunoblotting (Fig. 7A). Whole cell lysates of INSR siRNA-transfected hTCEpi cells were then subjected to immunoprecipitation by αIR3, C-19, or anti-INSRα (MA-20) antibodies. MA-20 does not recognize Hybrid-R [23], thus allowing identification of homodimeric INSR in hTCEpi cells. Immunoprecipitates were analyzed by western blotting with either anti-IGF-1R or anti-INSR antibodies (Fig. 7B). We found that INSR depletion was associated with significantly reduced homodimeric INSR in hTCEpi cells while the level of Hybrid-R and homodimeric IGF-1R remained unchanged. To dissect the subcellular distribution of IGF-1R homodimer and Hybrid-R, cytosolic and nuclear fractions of INSR siRNA-transfected hTCEpi cells were immunoprecipitated with antibodies against anti-IGF-1R (αIR3) or anti-INSR (C-19). No significant cross-contamination between each compartment was confirmed by immunoblotting with antibodies against GAPDH (cytosolic extract) and SP1 (nuclear extract). Immunoblots of IGF-1R showed a constant level of cytosolic and nuclear Hybrid-R (co-precipitated by C-19) in INSR-depleted hTCEpi cells while nuclear IGF-1R homodimer was undetectable in both conditions (Fig. 7C). Knockdown of INSR also resulted in an increase in total IGF-1R expression (Fig. S2A). Since Hybrid-R levels were not reduced, we confirmed specificity of the C-19 antibody. Western blotting of whole cell lysates and co-IP following knockdown of either the INSR or the IGF-1R demonstrated that C-19 antibody recognized INSR and not IGF-1R (Fig. S2). These results suggest that the presence of increased IGF-1R complexes with remaining INSR in cytosolic and nuclear fractions, resulting in preferential formation of Hybrid-R in hTCEpi cells.

### Potential Nuclear Targets of IGF-1R/INSR

To explore the functional relevance of nuclear IGF-1R, we identified the genomic DNA sequences to which IGF-1R bound in hTCEpi cells using a chromatin immunoprecipitation sequencing (ChIP-seq) assay. DNA fragments immunoprecipitated by IGF-1R (CST#3027) or INSR (C-19) were subjected to massively parallel DNA sequencing and bioinformatics analysis. Approximately 21.6 million (IGF-1R) and 14.2 million (INSR) reads were produced, of which about 15 million (IGF-1R) and 9.7 million (INSR) survived several rounds of filtering and were successfully aligned to the human reference genome hg19. To obtain statistically significant IGF-1R or INSR-enriched regions, the data set was analyzed with MACS [18] and PeakRanger [19] software, resulting in 179 (IGF-1R) and 174 (INSR) candidate peaks called by MACS (Fig. S4 and S6). The distribution of MACS peaks over the chromosome regions showed the fragments enriched by IGF-1R or INSR antibodies were located in chromosome 4, 10, 16, 21 and Y (Fig. S3 and S5). The shared peaks called by both MACS and PeakRanger generated 88 (IGF-1R) and 86 (INSR) final candidate peaks. The final candidate peaks were assigned to identify nearest genes, yielding 138 (IGF-1R) or 133 (INSR) known genes. We next passed these genes to the DAVID bioinformatics database [21], [22] for functional annotation. There were 52 (IGF-1R) and 31 (INSR) enriched DNA sequences being identified as known functional human genes, including 11 genes involved in cell proliferation/cell cycle control and 6 genes related to cell death and apoptosis (Table 2). GOTERM_BP term analysis showed that the majority of the most enriched terms describing biological processes affected by IGF-1R or INSR were associated with cell adhesion or positive regulation of transport, respectively (Table 3).

**Table 2:** Functional clustering of gene annotations using the DAVID resource.

	IGF-1R	INSR
Total Functional Genes Identified	52	31
*Involved in regulation of:*		
Cell proliferation/cell cycle progress	5	6
Cell death/apoptosis	5	1
Cell differentiation	6	1
Cell adhesion	6	3
Signal transduction	11	5
Cellular protein metabolic process	8	7
Cell communication	3	3

**Table 3:** Gene ontology clustering of gene annotations.

	Category^a^	Term	Count^b^	%^c^	P-value^d^
IGF-1R	*GOTERM_BP_FAT*	Cell-cell adhesion	5	9.6	3.1E-3
	*GOTERM_BP_5*	Neurogenesis	5	9.6	3.6E-2
	*GOTERM_BP_3*	Signal transduction	11	21.2	8.2E-2
INSR	*GOTERM_BP_ALL*	Positive regulation of secretion	3	9.7	8.3E-3
	*GOTERM_BP_ALL*	Cellular process	18	58.1	3.7E-2
	*GOTERM_BP_ALL*	Negative regulation of proliferation	3	9.7	7.6E-2

The enriched terms associated with the smallest p-value and with p<0.1 were shown.

a original database where the terms orient.

b genes involved in the term.

c percentage  =  involved genes/total genes.

d modified Fisher Exact p-value is adopted to measure the gene-enrichment in annotation terms.

## Discussion

While expression of IGF-1R and INSR have been previously reported in the corneal epithelium, the significant finding in this study lies in the first identification of the IGF-1R/INSR hybrid in human corneal epithelial cells. This is supported by reciprocal co-immunoprecipitation assays confirming the presence of Hybrid-R in whole lysates. Importantly, the Hybrid-R was detected at the plasma membrane, demonstrated by the ability of IGF-1 to induce phosphorylation of the receptor, and within the epithelial cell nucleus. While previous reports have investigated a role for IGF-1 in stimulating proliferation and migration in corneal epithelial cells, the mechanism by which IGF-1 stimulates proliferation is not well defined. Consistent with previous reports of IGF-1 as a potent mitogen in corneal epithelial cells, we found that IGF-1 stimulated Akt signaling and proliferation, which was effectively blocked by co-incubation with the insulin-like growth factor binding protein-3. Moreover, the use of a neutralizing antibody, αIR3, which has been previously shown to specifically recognize the IGF-1R homodimer, abrogated IGF-1 mediated proliferation, confirming that IGF-1 stimulated proliferation through activation of the IGF-1R as opposed to the Hybrid-R. The stimulatory effect of IGF-1 on corneal epithelial proliferation was through Akt signaling, since treatment with αIR3 did not affect ERK1/2 (MAPK family) activation (data not shown).

We have recently reported the nuclear localization of IGF-1R in an immortalized human corneal epithelial cell line and primary cultured corneal epithelial cells [14]. In this study we extended these previous findings to characterize the profile of the nuclear localized receptor. While we were readily able to detect the IGF-1R homodimer in whole cell lysates, of high significance in this study, we were only able to detect the Hybrid-R in the nucleus of corneal epithelial cells. The nuclear localization of the Hybrid-R was confirmed by reciprocal immunoprecipitation of nuclear extracts and the direct demonstration of colocalized Hybrid-R in the nucleus by confocal microscopy. The novel finding of a nuclear localizing Hybrid-R provides a potential mechanism underlying the trafficking of IGF-1R to the nucleus that was recently reported in cancer cells [7]. Since INSR, but not IGF-1R, possesses a putative nuclear localization sequence (NLS) [28], it is conceivable that the INSR escorts IGF-1R as a heterotetrameric hybrid complex to the nucleus through the importin system. Alternatively, sumoylation-mediated translocation of IGF-1R to the nucleus has recently been shown in DFB melanoma and human embryonic kidney cell lines, and the over-accumulation of SUMO conjugating enzyme (Ubc9) in the tumor nucleus is associated with elevated level of nuclear IGF-1R [8], [29]. Since IGF-1R sumoylation sites are conserved in the INSR, Hybrid-R may traffic into the nucleus through SUMO modification of either IGF-1R or INSR. It is unknown whether Ubc9 is over-expressed in the corneal epithelial nucleus, thus the potential for sumoylation to mediate nuclear localization of the Hybrid-R is of high interest to be investigated.

We have previously reported that IGF-1R localizes to the nucleus of corneal epithelial cells and in contrast to prior reports on nuclear localized IGF-1R using serum models [7], [8], we have been unable to deplete IGF-1R from the nucleus of corneal epithelial cells following growth factor deprivation. Similarly, subsequent IGF-1 ligand stimulation at the plasma membrane is not able to increase nuclear IGF-1R levels [14]. While we provide evidence that the Hybrid-R is phosphorylated at the plasma membrane, we have not yet established whether IGF-1 mediates nuclear trafficking of the IGF-1R or whether this receptor traffics to the nucleus independent of ligand. Intriguingly, extracellular ligand-independent nuclear trafficking has been identified for other growth factor receptors, including the FGFR1 and the EGFR [30], [31], [32]. Due to the presence of an abnormal transmembrane domain, the FGFR1 has been shown to undergo an integrative signaling pathway mediated by intracellular FGFs [32]. Similarly, upon appropriate stimulation such as DNA damage, the EGFR will translocate from the golgi to the nucleus in the absence of any ligand stimuli [33], [34], [35]. Thus, the mechanism underlying nuclear trafficking of Hybrid-R in corneal epithelial cells requires additional work to unravel.

The biological significance of the Hybrid-R has been implicated in human cancer and diabetes. Both IGF-1R and INSR are found over-expressed in a variety of human cancer types and are associated with malignant tumor progression [36]. In cells expressing both IGF-1R and INSR, Hybrid-Rs form by random assembly of half-receptors before insertion into the plasma membrane [4]. Over-expression of both IGF-1R and INSR in cancer cells leads to an over-expression of Hybrid-R as well; however, the mechanisms that regulate Hybrid-R formation are currently unknown. In efforts to deplete the Hybrid-R in our study, we utilized siRNA to reduce INSR expression. Surprisingly, reduced INSR expression resulted in an inhibition of INSR homodimer expression in whole cell lysates, but Hybrid-R was preserved (Fig. 7). The absence of a detectable decrease of Hybrid-R in the INSR-depleted hTCEpi cells is likely due to enrichment of Hybrid-R immunoprecipitates left in the lysates. Alternatively, knockdown of INSR also resulted in an increase in total IGF-1R expression in hTCEpi cells (Fig. S2A), which is consistent with the finding that the siRNA knockdown of INSR increases IGF-1R expression in human myeloma cells [37]. Increased IGF-1R may sequester remaining INSR to form Hybrid-R, leading to unchanged or slightly increased Hybrid-R in INSR-knockdown hTCEpi cells. The preferential formation of Hybrid-R under low INSR levels suggests that the Hybrid-R is a key regulatory protein that is essential for survival and maintenance of corneal epithelial cells.

The functional significance of the nuclear Hybrid-R represents a desirable area to be explored in all cell systems. In corneal epithelial cells, nuclear IGF-1R localizes to the chromatin bound fraction suggesting a role in gene regulation. Similarly, in cancer cells, recent reports indicate that nuclear IGF-1R may function to regulate gene expression as a transcriptional modulator [7], [8]. Our genome-wide ChIP-seq results, which were performed in the presence of low levels of IGF-1 in the growth media, confirmed the interaction of DNA with IGF-1R or INSR. Gene ontology analysis showed that the majority of the clustering genes potentially affected by IGF-1R were associated with the cell-cell adhesion process (Table 3). Previous studies have shown that at the plasma membrane, inhibition of IGF-IR results in suppression of adhesion and invasion in metastatic breast cancer. Similarly, a dominant negative mutant of the IGF-1R (486stop), which is secreted extracellularly, was shown to inhibit MDA-MB-435 and MDA-MB-231 cellular adhesion to laminin and collagen [38]. In addition, IGF-1R activation has been reported in a number of cell types to be responsible for the disruption of intercellular adhesion through the formation of multimolecular complexes containing components of the IGF-1R signaling pathway together with adherens junction proteins [39]. Nevertheless, a potential role for IGF-1R in regulating cellular adhesion at the transcriptional level warrants further investigation.

Hybrid-R has been shown to be abundantly expressed in adipose tissue and skeletal muscle of type 2 diabetic patients [40], [41], and is associated with lower insulin sensitivity. In contrast to stimulation by IGF-1, in our cell system, Hybrid-R was poorly activated by insulin. Since Hybrid-R responds poorly to insulin stimulation, an elevated level of Hybrid-R may impair insulin action by sequestering insulin receptors in a less responsive form [42]. The obvious clinical implication of Hybrid-R in the corneal epithelium relates to its potential roles in mediating ocular surface disease phenotypes in the diabetic eye. Importantly, diabetic keratopathy is a significant clinical complication arising from systemic disease, that can result in chronic and sometimes permanent vision loss [43]. Thus, the presence of Hybrid-R in the corneal epithelium has important roles in maintenance of the healthy cornea, as well as pathological changes resulting from diabetic disease.

The nuclear localization of the Hybrid-R adds another level of complexity underlying how IGF-1R and INSR signaling and trafficking contribute to both normal tissue homeostasis and the underlying pathobiology of disease. Further studies investigating changes in Hybrid-R expression and its subcellular distribution in the healthy and diabetic corneal epithelium are of great importance. Moreover, due to the high structural and functional homology between IGF-1R homodimer and Hybrid-R, the effect on both receptors should be carefully considered during therapeutic development.

## Supporting Information

Figure S1 **Characterization of homodimeric INSR phosphorylation in hTCEpi cells.** hTCEpi cells were starved for 24 h in KBM-2 culture medium and then stimulated with IGF-1 (100 ng/ml) or insulin (100 ng/ml) for 15 min. (A) Whole cell lysates were immunoblotted (IB) with anti-phospho-IGF-1R (Tyr1135), anti-IGF-1R, anti-phospho-INSR (Tyr1146), or anti-INSR as indicated. (B) WCL of hTCEpi cells were immunoprecipitated with antibody against INSR (MA-20). Immunoprecipitates were then immunoblotted with anti-INSR (C-19) or anti-PY20.
(TIF)

Figure S2 **Antibody against INSR (C-19) is specific to INSR.** (A) Control, INSR, or IGF-1R siRNA were transfected into hTCEpi cells. Two days after transfection, WCL were collected and subjected to (A) immunoblotting analysis with antibodies against INSR (C-19), IGF-1R (CST#3027), or β-actin (loading control). (B) WCL of hTCEpi cells were immunoprecipitated with antibody C-19. Immunoprecipitates were then immunoblotted with anti-INSR (C-19).
(TIF)

Figure S3 **Statistically significant enrichment of IGF-1R-bound chromosome regions.**
(TIF)

Figure S4 **The distribution of IGF-1R-enriched MACS peaks over chromosome regions.**
(TIF)

Figure S5 **Statistically significant enrichment of INSR-bound chromosome regions.**
(TIF)

Figure S6 **The distribution of INSR-enriched MACS peaks over chromosome regions.**
(TIF)
