# Supplementary figures and images for: Novel Nuclear Localization and Potential Function of Insulin-Like Growth Factor-1 Receptor/Insulin Receptor Hybrid in Corneal Epithelial Cells
Source: PLoS One. 2012 Aug 3;7(8):e42483. doi: 10.1371/journal.pone.0042483 (PMC3411736; doi:10.1371/journal.pone.0042483)

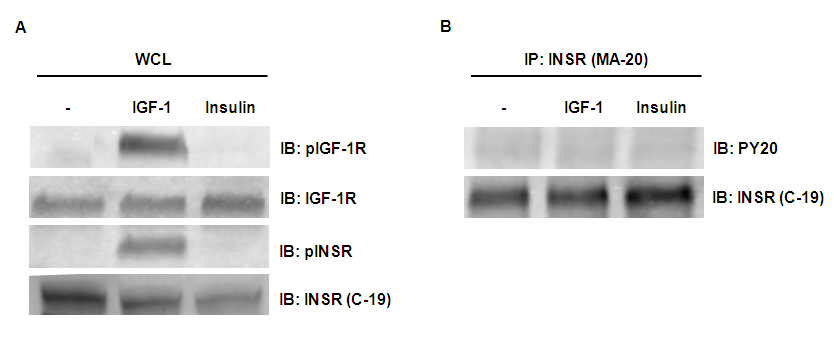

Supplement: Figure S1 — Characterization of homodimeric INSR phosphorylation in hTCEpi cells. hTCEpi cells were starved for 24 h in KBM-2 culture medium and then stimulated with IGF-1 (100 ng/ml) or insulin (100 ng/ml) for 15 min. (A) Whole cell lysates were immunoblotted (IB) with anti-phospho-IGF-1R (Tyr1135), anti-IGF-1R, anti-phospho-INSR (Tyr1146), or anti-INSR as indicated. (B) WCL of hTCEpi cells were immunoprecipitated with antibody against INSR (MA-20). Immunoprecipitates were then immunoblotted with anti-INSR (C-19) or anti-PY20. (TIF) [file pone.0042483.s001.tif]

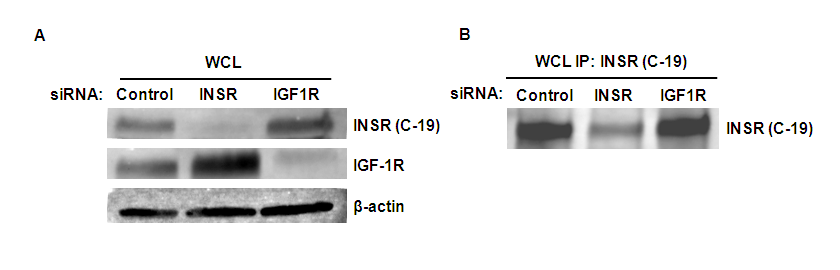

Supplement: Figure S2 — Antibody against INSR (C-19) is specific to INSR. (A) Control, INSR, or IGF-1R siRNA were transfected into hTCEpi cells. Two days after transfection, WCL were collected and subjected to (A) immunoblotting analysis with antibodies against INSR (C-19), IGF-1R (CST#3027), or β-actin (loading control). (B) WCL of hTCEpi cells were immunoprecipitated with antibody C-19. Immunoprecipitates were then immunoblotted with anti-INSR (C-19). (TIF) [file pone.0042483.s002.tif]

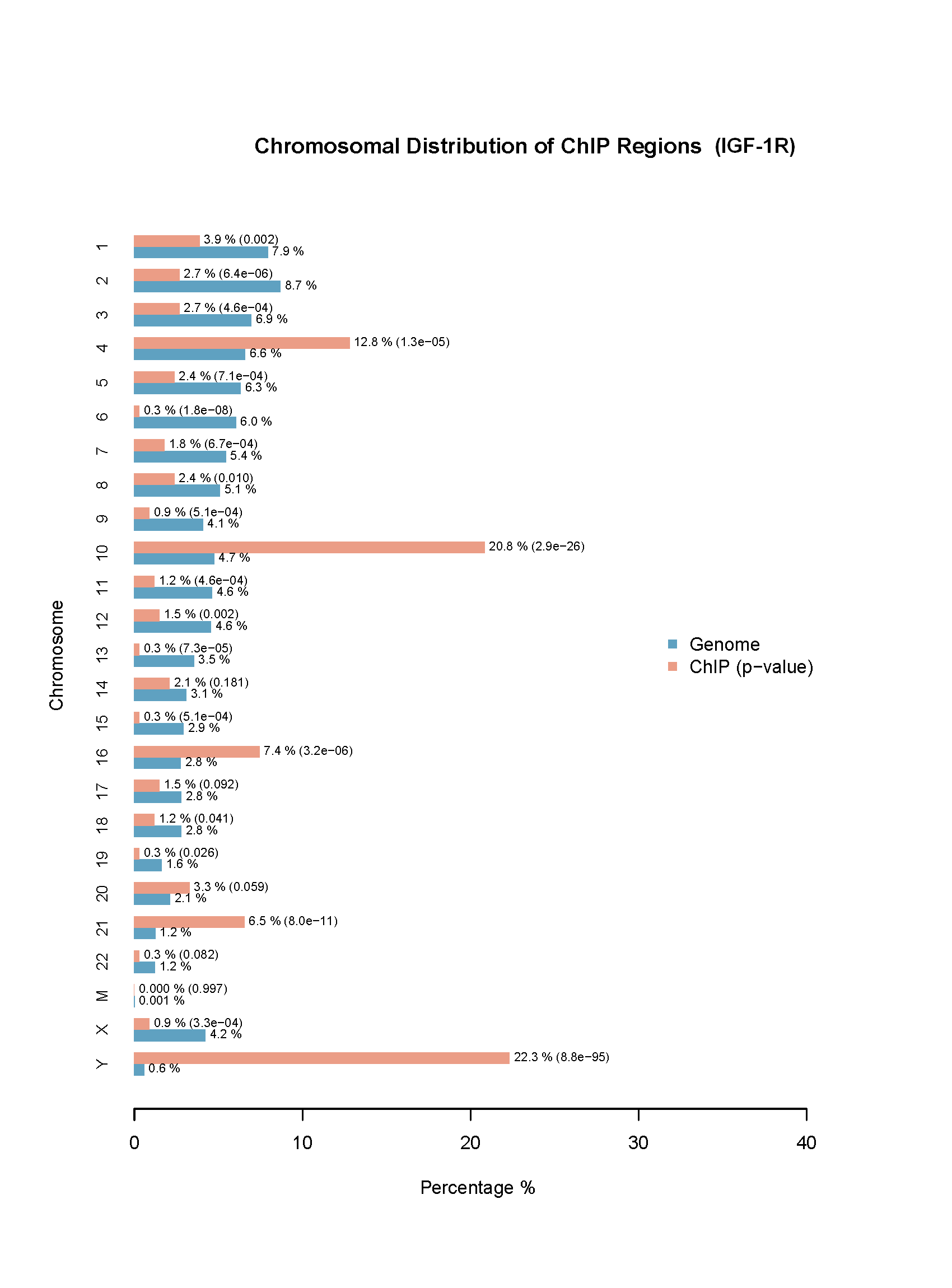

Supplement: Figure S3 — Statistically significant enrichment of IGF-1R-bound chromosome regions. (TIF) [file pone.0042483.s003.tif]

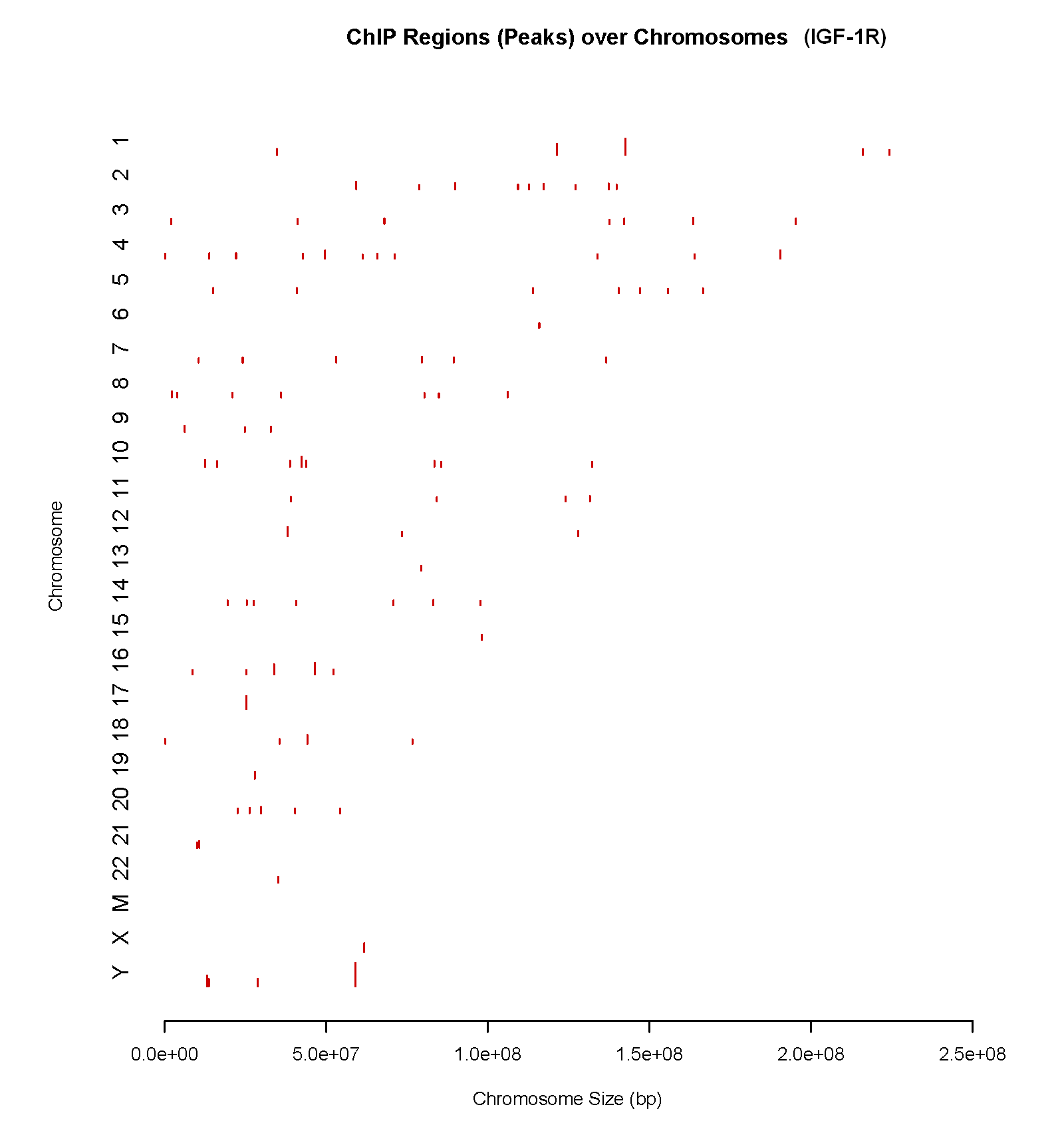

Supplement: Figure S4 — The distribution of IGF-1R-enriched MACS peaks over chromosome regions. (TIF) [file pone.0042483.s004.tif]

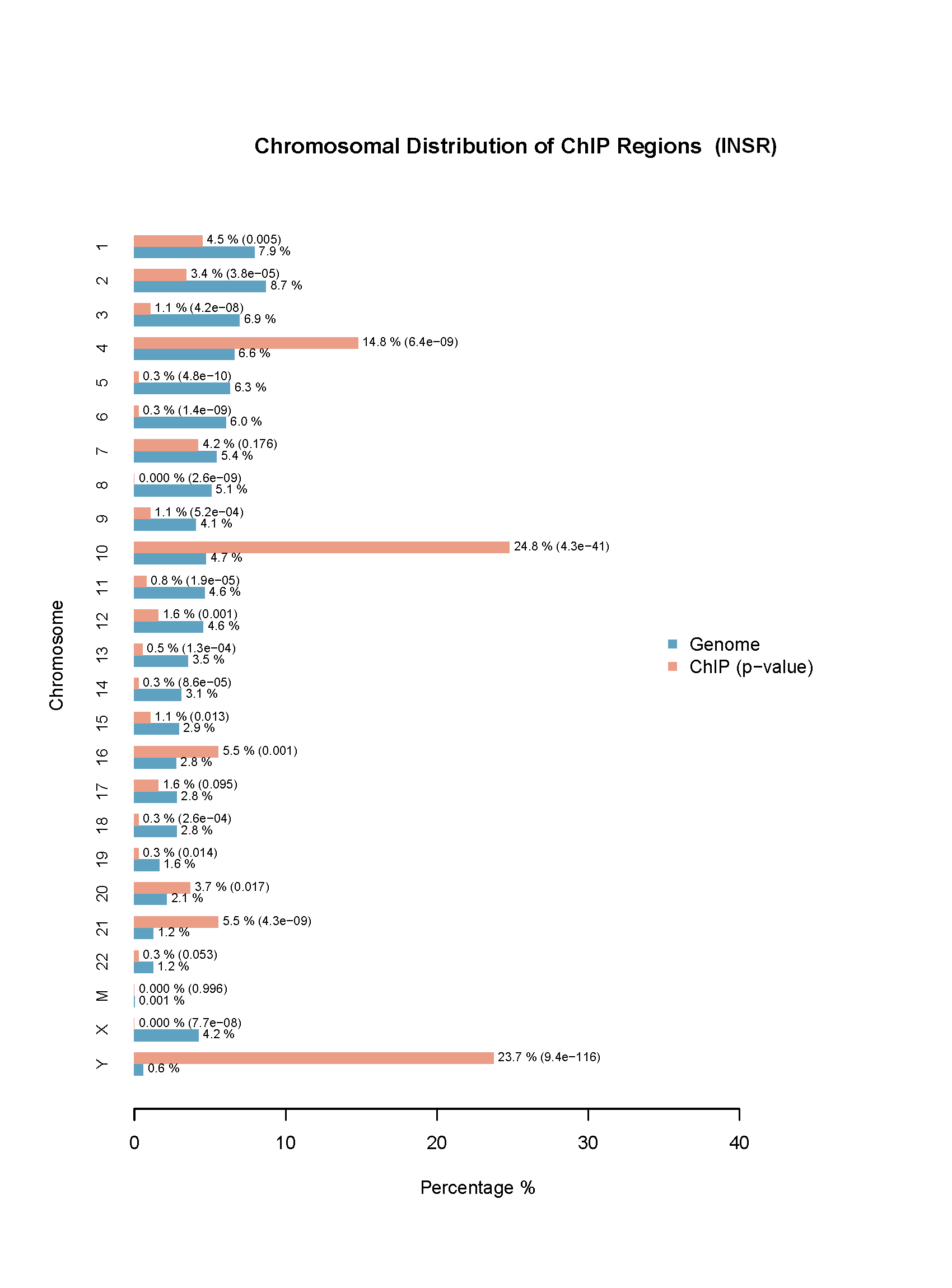

Supplement: Figure S5 — Statistically significant enrichment of INSR-bound chromosome regions. (TIF) [file pone.0042483.s005.tif]

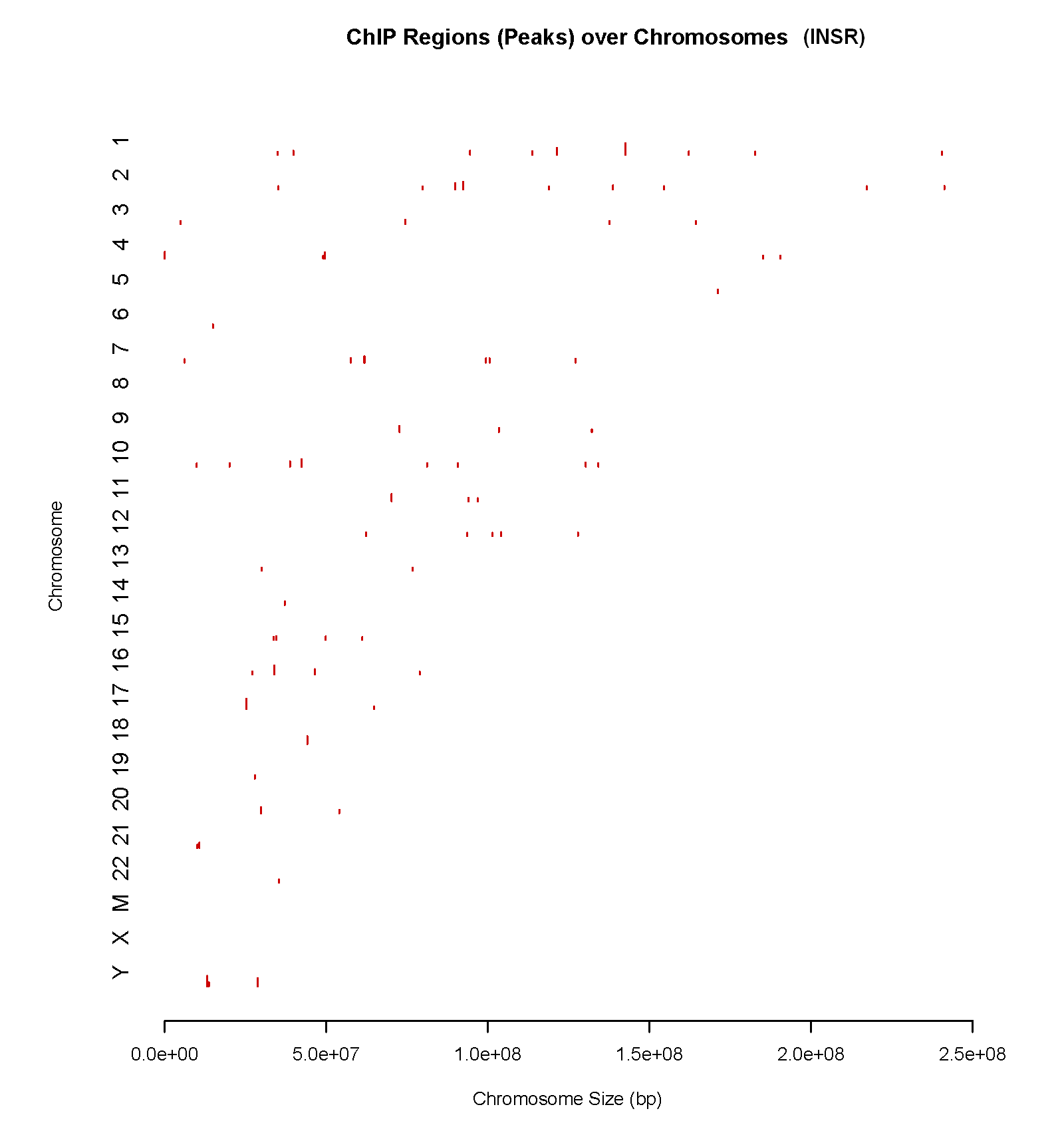

Supplement: Figure S6 — The distribution of INSR-enriched MACS peaks over chromosome regions. (TIF) [file pone.0042483.s006.tif]
